# The short-term effectiveness of coronavirus disease 2019 (COVID-19) vaccines among healthcare workers: a systematic literature review and meta-analysis

**DOI:** 10.1017/ash.2021.195

**Published:** 2021-10-21

**Authors:** Alexandre R. Marra, Takaaki Kobayashi, Hiroyuki Suzuki, Mohammed Alsuhaibani, Bruna Marques Tofaneto, Luigi Makowski Bariani, Mariana de Amorim Auler, Jorge L. Salinas, Michael B. Edmond, João Renato Rebello Pinho, Luiz Vicente Rizzo, Marin L. Schweizer

**Affiliations:** 1 Department of Internal Medicine, University of Iowa Carver College of Medicine, Iowa City, Iowa, United States; 2 Instituto Israelita de Ensino e Pesquisa Albert Einstein, Hospital Israelita Albert Einstein, São Paulo, Brazil; 3 Center for Access & Delivery Research & Evaluation (CADRE), Iowa City Veterans’ Affairs Health Care System, Iowa City, Iowa, United States; 4 Department of Pediatrics, College of Medicine, Qassim University, Qassim, Saudi Arabia; 5 Albert Einstein Medical College, São Paulo, Brazil; 6 West Virginia University School of Medicine, Morgantown, West Virginia, United States; 7 Special Techniques Laboratory, Hospital Israelita Albert Einstein, São Paulo, Brazil

## Abstract

**Objective:**

Healthcare workers (HCWs) are at risk of COVID-19 due to high levels of SARS-CoV-2 exposure. Thus, effective vaccines are needed. We performed a systematic literature review and meta-analysis on COVID-19 short-term vaccine effectiveness among HCWs.

**Methods:**

We searched PubMed, CINAHL, EMBASE, Cochrane Central Register of Controlled Trials, Scopus, and Web of Science from December 2019 to June 11, 2021, for studies evaluating vaccine effectiveness against symptomatic COVID-19 among HCWs. To meta-analyze the extracted data, we calculated the pooled diagnostic odds ratio (DOR) for COVID-19 between vaccinated and unvaccinated HCWs. Vaccine effectiveness was estimated as 100% × (1 − DOR). We also performed a stratified analysis for vaccine effectiveness by vaccination status: 1 dose and 2 doses of the vaccine.

**Results:**

We included 13 studies, including 173,742 HCWs evaluated for vaccine effectiveness in the meta-analysis. The vast majority (99.9%) of HCWs were vaccinated with the Pfizer/BioNTech COVID-19 mRNA vaccine. The pooled DOR for symptomatic COVID-19 among vaccinated HCWs was 0.072 (95% confidence interval [CI], 0.028–0.184) with an estimated vaccine effectiveness of 92.8% (95% CI, 81.6%–97.2%). In stratified analyses, the estimated vaccine effectiveness against symptomatic COVID-19 among HCWs who had received 1 dose of vaccine was 82.1% (95% CI, 46.1%–94.1%) and the vaccine effectiveness among HCWs who had received 2 doses was 93.5% (95% CI, 82.5%–97.6%).

**Conclusions:**

The COVID-19 mRNA vaccines are highly effective against symptomatic COVID-19, even with 1 dose. More observational studies are needed to evaluate the vaccine effectiveness of other COVID-19 vaccines, COVID-19 breakthrough after vaccination, and vaccine efficacy against new variants.

The first coronavirus disease 19 (COVID-19) vaccine was authorized for emergency use by the US Food and Drug Administration on December 11, 2020, for prevention against infection in individuals 16 years or older who are healthy or have stable chronic medical conditions and were eligible for participation in the trial. That mRNA vaccine demonstrated an efficacy of 95%.^1^ Subsequently, 8 more vaccines have been authorized for full use.^2^

During the first year of the COVID-19 pandemic, healthcare workers (HCWs) were at high risk of acquiring COVID-19.^3,4^ Compared to the community, some studies have shown that frontline HCWs had >10 times higher risk of testing positive for severe acute respiratory coronavirus virus 2 (SARS-CoV-2) and that those who reported that they had inadequate access to personal protective equipment (PPE) had a 23% higher risk.^3,4^ Also, compared to HCWs reporting adequate PPE who did not care for patients with COVID-19, workers caring for patients with documented COVID-19 had a nearly 5 times higher risk of testing positive if they had adequate PPE and a nearly 6 times higher risk if they had inadequate PPE.^4^

Over the past few months, research studies have contributed a large amount of data from different institutions on COVID-19 vaccine roll-out, making available real-world data on short-term vaccine effectiveness.^5,6^ These vaccines are effective for a wide range of COVID-19–related outcomes, a finding consistent with that of the randomized trials,^1,7^ and they show benefits in HCWs.^8,9^

We reviewed the literature on the impact of the short-term effectiveness of COVID-19 vaccines among HCWs to prevent laboratory-confirmed COVID-19. Pooling the results of published studies allows for more precise estimates of vaccine effectiveness and for subset analyses, such as evaluating the effectiveness of the vaccine against symptomatic COVID-19 and asymptomatic COVID-19 separately.

## Methods

### Systematic literature review and inclusion and exclusion criteria

This review was conducted according to the Preferred Reporting Items for Systematic Reviews and Meta-Analysis (PRISMA) statement^10^ and the Meta-analysis of Observational Studies in Epidemiology (MOOSE) guidelines.^11^ This study was registered on Prospero (https://www.crd.york.ac.uk/PROSPERO/) on May 21, 2021 (registration no. CRD42021255589). Institutional review board approval was not required. We applied the following inclusion criteria: original research manuscripts; articles published in peer-reviewed scientific journals; studies involving vaccinated and unvaccinated HCWs; studies conducted in acute-care settings or nursing homes that evaluated the effectiveness of COVID-19 vaccine in HCWs after phase 3 clinical trials; and studies with an observational design. The literature search was limited to the period from December 2019 to June 11, 2021. Randomized clinical trials (phase 3), editorials, commentaries, and published studies from non–peer-reviewed studies (eg, MedRxiv) were excluded. Studies in which there was no comparison between vaccinated and unvaccinated HCWs, and those in which no vaccine effectiveness data were published were also excluded.

### Search strategy

**Fig. 1.**
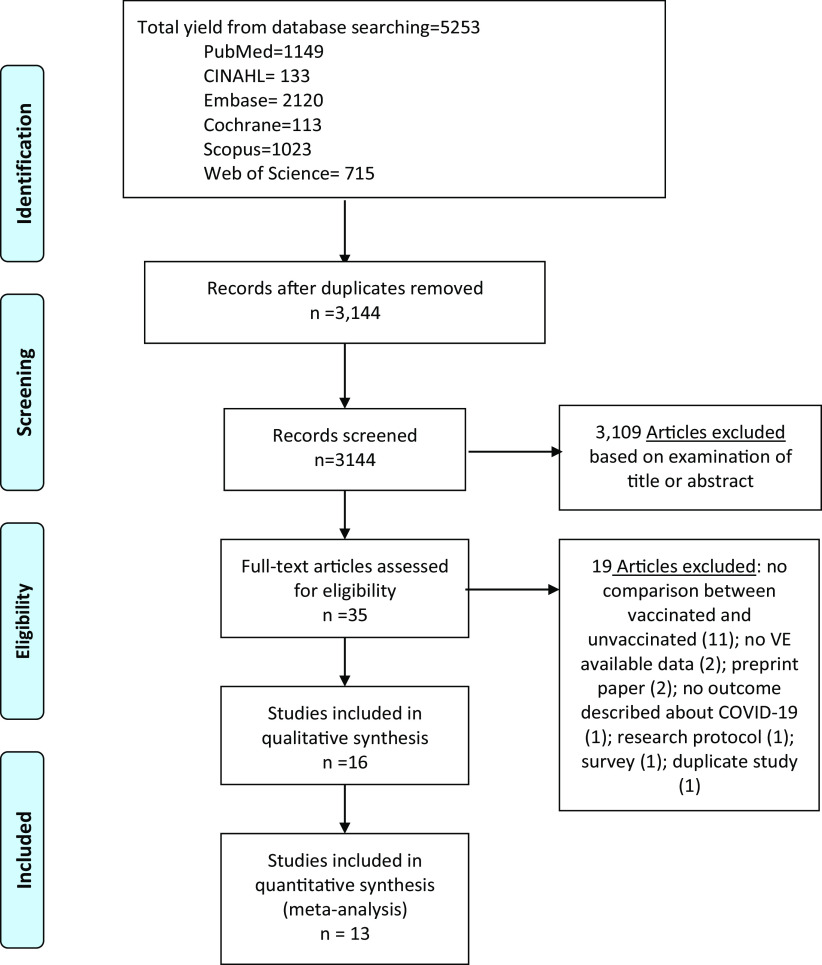
Literature search for articles on COVID-19 vaccine effectiveness among healthcare workers.

We performed literature searches in PubMed, Cumulative Index to Nursing and Allied Health (CINAHL), Embase (Elsevier Platform), Cochrane Central Register of Controlled Trials, Scopus (which includes EMBASE abstracts), and Web of Science. The entire search strategy is described in Supplementary Appendix 1. We reviewed the reference lists of retrieved articles to identify studies that were not identified from the preliminary literature searches. After applying exclusion criteria, we reviewed 35 papers; 16 of these met the inclusion criteria and were included in the systematic literature review (Fig. 1).

### Data abstraction and quality assessment

Titles and abstracts of all articles were screened to assess whether they met inclusion criteria. The reviewers (A.R.M., H.S., M.A.A., and T.K.) abstracted data from each article. Reviewers resolved disagreements by consensus.

The reviewers abstracted data on study design, population and setting, and the time (in days) of vaccination status (1 dose or 2 doses). The FDA recommends defining the COVID-19 end point as virologically confirmed SARS-CoV-2 infection accompanied by symptoms.^12^ For that reason, we defined the primary outcome as symptomatic COVID-19. For our stratified analysis, we also investigated symptomatic and asymptomatic COVID-19 combined and only asymptomatic COVID-19.

We also collected information about the incidence rate ratio (IRR), the rate reduction (RRed), the hazard ratio (HR), the relative risk (RR), the odds ratio (OR) with 95% confidence interval (CI), the vaccine effectiveness with 95% CI, and the statistical analysis performed by each included study. We also assessed the potential risk of bias for each study using the Downs and Black scale.^13^ Reviewers followed all questions from this scale as written except for question 27 (a single item on the power subscale scored 0 to 5), which was changed to a yes or no. Also, 2 authors performed component quality analyses independently, reviewed all inconsistent assessments, and resolved disagreements by consensus.^14^

### Statistical analysis

To meta-analyze the extracted data, we calculated the pooled diagnostic odds ratio (DOR) with the 95% confidence interval for symptomatic COVID-19 between vaccinated and unvaccinated HCWs. Vaccine effectiveness was estimated as 100% × (1 − DOR). We also performed stratified analyses with the association between the HCW vaccination status (ie, 1 dose or 2 doses) and COVID-19 symptomatic status (ie, symptomatic, symptomatic and asymptomatic, or asymptomatic). If the study reported 2 doses, we calculated the vaccine effectiveness after the second dose. If the study reported only 1 dose, we calculated the vaccine effectiveness after the first dose. If the study reported >1 vaccine effectiveness rate with different postvaccination periods, we used the vaccine effectiveness of the longest period. We performed statistical analyses using R version 4.1.0 software with the mada package version 0.5.4.^15^ Analogous to the meta-analysis of the odds ratio methods for the DOR, an estimator of random-effects model following the approach of DerSimonian and Laird is provided by the mada package.^15^ For our meta-analysis of estimates of COVID-19 vaccine effectiveness, we used a bivariate random effects model, adopting a similar concept of performing the diagnostic accuracy, which enabled simultaneous pooling of sensitivity and specificity with mixed-effect linear modeling while allowing for the trade-off between them.^16,17^ Heterogeneity between studies was evaluated using I^2^ estimation and the Cochran Q statistic test.

## Results

### Characteristics of included studies

Overall, 16 studies met the inclusion criteria^18–33^ and were included in the final review (Table 1). All of these studies were nonrandomized: 8 were retrospective cohort studies,^18,19,21–23,26,30,33^ 6 were prospective cohort studies,^20,25,28,29,31,32^ and 2 were case–control studies.^24,27^ All of the studies evaluated the Pfizer/BioNTech mRNA COVID-19 vaccine,^18–33^ 2 studies also analyzed the Moderna mRNA COVID-19 vaccine,^27,31^ and another study also analyzed the AstraZeneca COVID-19 vaccine^25^ (but this study was not considered in the vaccine effectiveness analysis). No study evaluated the vaccine effectiveness for the Johnson & Johnson/Janssen vaccine. Nearly all HCWs (99.9%) were vaccinated with the Pfizer/BioNTech COVID-19 mRNA vaccine.^18–33^

**Table 1.** Summary of Characteristics of Studies Included in the Systematic Literature Review

First Author, Year, Location	COVID-19 Vaccine	Study Design	Study Period, Duration and Date	No. of HCWs and Characteristics	Follow-Up Time After the First Dose, Days, No. [%]	Follow-up Time After the Second Dose, Days, No. [%]	COVID-19 S/A (N)		COVID-19 S (N)		IRR, RRed, HR, RR’, or OR (95% CI), and VE (95% CI)	Statistical Analysis Performed	D&B Score (max. score, 28)
							First Dose	Second Dose	First Dose	Second Dose			
Amit 2021, Israel	Pfizer/BioNTech	Retrospective cohort	1 mo [Dec 19, 2020–Jan 24, 2021]	9,109 (not stated)	1–14 d after first dose N = 7,214 [79.0%]	Received the second dose on day 21 or 22 after the first dose N = 6,037 [66.0%]	89	7	60	2	Two COVID-19 doses: COVID-19 (S/A): RRed = 86% (70%–94%) COVID-19 (S) RRed = 94% (76%–99%)	Rate ratios and the 95% were estimated using Poisson regression with logarithm of the community exposure as offset. The adjusted estimates were subtracted from 1 to obtain the rate reductions	20
Angel 2021, Israel	Pfizer/BioNTech	Retrospective cohort	2 mos [Dec 20, 2020–Feb 25, 2021]	6,710 (65% female; age, mean y = 44.3 [SD, ±12.5]; 19% physicians, and 25.5% nurses)	7–28 d after first dose N=5,953 [88.7%]	>7 d after second dose N = 5,517 [82.2%]	55 (17 asymptomatic)	27 (19 asymptomatic)	38	8	2 COVID-19 vaccine doses: Adjusted IRR = 0.03 (0.01–0.06) VE = 97.0% (94.0%–99.0%)	Multivariable Poisson regression [covariates: age, sex, employment sector, exposure risk, and the no. of PCR tests for each healthcare worker in the period]	22
Bianchi 2021, Bari, Italy	Pfizer/BioNTech	Prospective cohort	1 mo [Dec 27, 2020–Jan 31, 2021]	2,034 (57.8% female; age, mean y = 44.4 [SD, ±12.6]; 24.3% physicians, and 75.7% other)	14–20 and 21–27 days after first dose	≥7 d after second dose	NR	54 (vaccinated HCWs but first or second dose not stated)	NR	37 (vaccinated HCWs but but first or second dose not stated)	COVID-19 (S+A): VE = 61.9% (19.2%–82.0%) during 14–20 d after the first dose; VE = 87.9% (51.7%–97.0%) during the 21–27 d after the first dose and VE = 96.0% (82.2%–99.1%) ≥7 d after the second dose	Survival curves for the vaccinated and unvaccinated groups were plotted using Kaplan-Meier estimator. The IRR was calculated. VE defined as 1 − RR, the 95% CI were estimated	21
Cavanaugh* 2021, Kentucky, US	Pfizer/BioNTech	Retrospective cohort	2 mos [vaccination days: Jan 10, Jan 31, and Feb 21; outbreak: March 1]	116 (not stated)	NR	>14 d after second dose N=61 [52.6%]	16	4	15	2	COVID-19 S/A: RR = 4.1 (1.5–11.6); VE = 75.9% (32.5%–91.4%) COVID-19: RR = 7.8 (1.9–32.4); VE = 87.1% (46.4%–96.9%)	Defined VE as 1 − RR of fully vaccinated vs unvaccinated ×100	17
Fabiani 2021, Treviso, Italy	Pfizer/BioNTech	Retrospective cohort	3 mos [Dec 27, 2020–Mar 24, 2021]	6,423 (56.5% female; age, mean y = 47.1 [SD, ±10.8]; 22.9% physicians, and 56.5% nurses)	0–14, 14–21, and ≥21 d after first dose N=147 (2.3%)	≥7 d after second dose N = 5,186 (80.7%)	2 (≥21 after first dose)	2	3 (1 asymptomatic; ≥21 after first dose)	4 (2 asymptomatic)	Adjusted VE for COVID-19 S: VE = 93.7% (50.8%–99.2%) ≥7 d after the second dose Adjusted VE for COVID-19 S: VE = 65.9% (−171% to 95.7%) during ≥21 d after the first dose	Multivariable Cox proportional hazard model, [covariates: sex, age group, professional category, work context, and week of exposure]. Adjusted HR were used to calculate VE as [(1-HR)x100]	22
Garvey 2021, Birmingham, UK	Pfizer/BioNTech	Retrospective cohort	2 mos [Dec 12, 2020–Feb 23, 2021]	∼30,000 (not stated)	>10 d after the first dose N=25,335 [of 30,000 in the work force]	NR	NR	NR	178	NR	Adjusted HR: 0.24 (95% CI, 0.20–0.28)	Multivariate logistic and weighted Cox regression models	13
Grass-Valenti 2021, Alicante, Spain	Pfizer/BioNTech	Case-control	2 weeks [Jan 27, 2021–Feb 7, 2021]	268 (77.6% female; 13.1% physicians, 39.9% nurses, 47% other)	>12 d after the first dose	NR	NR	NR	39	NR	Adjusted VE for COVID-19 S: VE = 52.6% (1.1%–77.3%)	Logistic regression model and the adjusted OR were used to calculate VE as [(1 − OR)×100]	22
Hall 2021, UK	Pfizer/BioNTech (A) and AstraZeneca (B)	Prospective cohort	2 mos [Dec 7, 2020–Feb 5, 2021]	23,324 (84% female; age, median y = 46 [IQR, 36.0–54.1]; 10.8% physicians, and 42.1% nurses)	>21 d after first dose N=20,641 [89.0%]; 19,384 [94.0%] of vaccine 1, and 1,252 [6.0%] of vaccine	>7 d after second dose N=1,607 [8.0%]; 1,605 [99.9%] of vaccine 1, and 2 [0.1%] of vaccine 2	977	3	71	NR	2 COVID-19 vaccine doses (A): Adjusted HR = 0.15 (0.04–0.26) VE = 85.0% (74.0%–96.0%) 1 COVID-19 vaccine dose (A): Adjusted HR = 0.30 (0.15–0.45) VE = 70.0% (55.0%–85.0%)	Mixed-effects multivariable logistic regression models (with hospital site as a random effect) and Poisson distribution	23
Jones* 2021, UK	Pfizer/BioNTech	Retrospective cohort	2 weeks [Jan 18, 2021–Jan 31, 2021]	∼9,000 weekly on site (no. of COVID-19 tests performed among HCWs both vaccinated and unvaccinated)	>12 d after first dose N=20,641 [89.0%]; 19,384 [94.0%] of vaccine 1, and 1,252 [6.0%] of vaccine	NR	13 + tests (HCWs <12 d postvaccine; and 4 + tests (HCWs <12 d postvaccine)	NR	NR	NR	4-fold decrease in the risk of asymptomatic COVID-19 among HCWs >12 d after vaccination	Fisher exact test	13
Pilishvili 2021, 25 US states	Pfizer/BioNTech (A) and Moderna (B)	Case-control	3 mos [Jan 2021 – Mar 2021	1,843 [623 case patients and 1,220 controls; 84% vs 82% females, respectively; age, median y = 38 [range, 19–69] for cases, and 37 [range, 19–76] for controls; 10.8% physicians, and 42.1% nurses)	>14 d after the first dose through day 6 after the second dose N = not clear	≥7 d after second dose N = 1,201 [65.2%]	NR	NR	NR	19 (*receivved ≥1 dose before test date)	2 COVID-19 vaccine doses (A and B): VE = 93.5% (86.5%–96.9%) 1 COVID-19 vaccine dose (A and B): VE = 81.7% (74.3%–86.9%)	Conditional logistical regression was used to estimate matched odds ratios [covariates: age, race/ethnicity, and presence of underlying conditions]	20
Pryor 2021, Richmond, VA	Pfizer/BioNTech	Prospective cohort	2 mos [Dec 16, 2020–Feb 12, 2021]	13,346 (not stated)	14 d after the first vaccine dose, N = 9,181 (69%)	NR	NR	NR	27	NR	1 COVID-19 vaccine dose: Adjusted OR = 0.02 (0.015–0.033) VE = 98.0% (96.7%–98.5%)	OR to determine VE	15
Sansone* 2021, Brescia, Italy	Pfizer/BioNTech	Prospective cohort	2.5 mos [Jan 25, 2021–Apr 13, 2021]	8,851 (not stated)	NR	≥7 d after second dose N = 6,904 [78.0%]	NR	40 (25 asymptomatic)	NR	15	Cumulative daily incidence of COVID-19 (per 10,000 people) among vaccinated and unvaccinated HCWs	OR (95% CI)	17
Swift 2021, Rochester, Minnesota, US	Pfizer/BioNTech	Retrospective cohort	3 mos [Jan 1, 2021– Mar 31, 2021]	71,152 (70.2% female; age, mean y = 41)	>14 d from first dose and ≤14 d from second dose N = 4,058 [5.7%]	>14 d after second dose N = 45,162 [63.5%]	997	30	876	22	2 COVID-19 vaccine doses: Adjusted IRR = 0.032 (0.022–0.047), VE = 96.8% (95.3%–97.8%); 1 COVID-19 vaccine dose: Adjusted IRR = 0.219 (0.180–0.267), VE = 78.0% (71.1%–82.0%)	Adjusted logistic regression model [covariates: age, gender, region, job and week of vaccination] with Poisson distribution	22
Tang 2021, Memphis, Tennessee, US	Pfizer/BioNTech	Prospective cohort	3 mos [Dec 17, 2020–Mar 20, 2021]	5,217 (vaccinated group: 66.0% female, 88.7% aged <65 y; unvaccinated group: 58.3% female, 84.4% aged <65 y)	≥12 d after first dose and before the second dose N=NR	≥7 d after second dose No. NR	17 (10 asymptomatic)	6 (6 asymptomatic)	7	0	COVID-19 (S+A): IRR = 0.04 (0.02–0.09) ≥7 d after second dose; COVID-19 (A): IRR = 0.10 (0.04–0.22) ≥7 d after second dose; and COVID-19 (S): No positive symptomatic case ≥7 d after the second dose	Survival curves for the vaccinated and unvaccinated groups were plotted using Kaplan-Meier estimator. The IRR was calculated.	18
Thompson 2021, Arizona, Florida, Minnesota, and Oregon, US	Pfizer/BioNTech (A) and Moderna (B)	Prospective cohort	3 mos [Dec 14, 2020–Mar 13, 2021]	3,950 (62.1% female; 71.9% aged 18–49 y; 21.1% physicians, 33.8% nurses)	≥14 d after the first dose and before the second dose [75% received ≥1 dose of vaccine; 477 [12.1%] received their first dose and had not received their second dose	≥14 days after second dose N=2,479 [62.8%] received both recommended mRNA vaccine doses	NR	NR	161	3	A and B for 2 COVID-19 vaccine doses: VE = 90.0% (68.0%–97.0%); A and B for 1 COVID-19 vaccine dose: VE = 80.0%	Adjusted logistic regression [covariates: sex, age, ethnicity and occupation] and Cox proportional hazard models	21
Walsh, 2021, Dublin, Ireland, UK	Pfizer/BioNTech	Retrospective cohort	2 mos [Dec 29, 2020–Feb 22, 2021]	4,458 (not stated)	0–7; 8–14;15–21; 22–30; and 39 days after first dose N = 3,805 (85.0%)	NR	77 (35 asymptomatic and 4 not documented)	NR	38	NR	Positivity rates between the vaccinated and unvaccinated groups differed significantly with 5.8% of the vaccinated cohort testing COVID-19 positive vs 25.6% of those tested in the unvaccinated cohort OR, 0.18 (95% CI, 0.13–0.25)	OR (95% CI)	14

* Genomic investigation about the new variants: (Cavanaugh 2021: R.1 lineage variant; Jones 2021: B.1.1.7 [alpha] variant; Sansone 2021: B.1.1.7 [α] variant)

Note. S/A, symptomatic and asymptomatic; S, symptomatic; SD, standard deviation; IQR, interquartile range; IRR, incidence rate ratio; RRed, rate reduction; HR, hazard ratio; RR’, relative risk; OR, odds ratio; CI, confidence interval; VE, vaccine effectiveness; D&B, Downs and Black scale; NR, not reported, N, no. reported.

Most of the studies included in our review were conducted in the United States (6 studies)^21,27,28,30–32^; 3 studies were performed in Italy^20,22,29^; 3 were performed in the United Kingdom^23,25,26^; 2 studies were performed in Israel^18,19^; 1 was conducted in Spain^24^; and 1 was conducted in Ireland.^32^ All studies were performed between December 2020 and April 2021.^18–33^

Overall, we included 195,801 HCWs in the qualitative analysis. Moreover, 6 studies evaluated vaccine effectiveness >7 days after the second dose,^19,20,22,25,27,29^ 3 studies evaluated vaccine effectiveness >14 days after the second dose,^21,30,32^ 1 study evaluated vaccine effectiveness after HCWs received the second dose on day 21 or day 22 after the first dose,^18^ and the other 6 studies did not report the time the vaccine was considered effective.^23,24,26,28,31,33^ Of the HCWs included that received the first dose, 6 studies evaluated the effectiveness of COVID-19 vaccine >14 days after the first dose,^20,22,27,28,30,32^ 2 studies evaluated the vaccine effectiveness >12 days after the first dose,^24,26^ 1 study evaluated the vaccine effectiveness >10 days after the first dose,^23^ and 1 study evaluated >21 days after the first dose.^20^ Also, 1 study evaluated vaccine effectiveness from day 1 to day 14 after the first dose.^18^ Another study evaluated vaccine effectiveness from day 7 to day 28 after the first dose,^21^ and another study evaluated vaccine effectiveness up 39 days after the first dose: 0−7 days, 8−14 days, 15−21 days, 22−30 days, and 39 days.^33^ Furthermore, 3 studies did not report the period after vaccination^27,29^ and 5 studies reported asymptomatic cases in vaccinated and unvaccinated HCWs.^19,22,25,30,31^

The studies we reviewed varied regarding the reportage of the infection rates and the type of statistical analyses performed. To determine vaccine effectiveness, 4 studies used Poisson distribution for adjusted logistic regression,^18,19,25,30^ 3 studies used adjusted regression and Cox proportional hazard models,^22,23,32^ and 3 studies used unadjusted odds ratio calculation.^28,29,33^ In addition, 2 studies used conditional logistical regression,^24,27^ 2 studies used survival curves for the vaccinated and unvaccinated groups using Kaplan-Meier,^20,31^ 1 study used the Fisher exact test,^26^ and 1 study used the risk ratio calculation to determine the vaccine effectiveness.^21^

Among the studies we reviewed, genomic surveillance detection of the new SARS-CoV-2 B.1.1.7 or α variant^26,29^ was conducted in 2 studies, and 1 outbreak study identified a new SARS-CoV-2 variant (R.1 lineage variant).^21^ Genomic surveillance was not performed in most of the studies we reviewed.^18–20,22–25,27,28,30–33^

Among the 3 studies that reported the presence of underlying medical conditions,^25,27,32^ 1 study showed that 75% of participants had no underlying medical condition,^25^ 1 study showed that ∼70% had no medical condition but that ∼30% had at least 1 chronic condition.^32^ In 1 case–control study, 75% of the case patients (symptomatic COVID-19) and the controls (non–COVID-19 patients) had at least 1 underlying condition or risk factor associated with increased risk for severe COVID-19.^27^ Proportions of immunocompromised HCWs were reported in only 2 studies with a rate at 2%^25^ to 5%.^27^ None of the included studies reported rates of adverse events after vaccination.^18–33^

Regarding the quality assessment scores of the 16 included studies, 9 studies were considered good quality (19–23 of 28 possible points) on the Downs and Black quality tool,^18–20,22,24,25,27,30,32^ 5 studies were considered fair quality (14–18 points),^21,28,29,31,33^ and 2 studies were considered poor quality (<14).^23,26^

### Results pooled by each COVID-19 vaccination dose and COVID-19 status

The review included 13 studies in which 173,742 HCWs were evaluated for vaccine effectiveness and were included in the meta-analysis.^19–25,27–31,33^ The pooled DOR for symptomatic COVID-19 among HCWs vaccinated with at least 1 dose was 0.072 (95% CI, 0.028–0.184), with an estimated the vaccine effectiveness of 92.8% (95% CI, 81.6%–97.2%). Among 13 studies, 7 studies evaluated vaccine effectiveness of 2 doses in HCWs.^19–22,27,29,30^ The pooled DOR for this group of studies was 0.065 (95% CI, 0.024–0.175) and the estimated vaccine effectiveness was 93.5% (95% CI, 82.5%–97.6%). Also, 6 studies evaluated vaccine effectiveness of 1 dose of vaccine in HCWs.^19,22,27,28,30,32^ The pooled DOR for these studies was 0.179 (95% CI, 0.059–0.539) and the estimated vaccine effectiveness was 82.1% (95% CI, 46.1%–94.1%).

Stratifying the analysis for studies reporting both symptomatic and asymptomatic COVID-19, 10 studies evaluated vaccine effectiveness among vaccinated HCWs who had received 1 and 2 doses.^19–23,25,29–31,33^ The pooled DOR for this group of studies was 0.082 (95% CI, 0.030–0.223) and the estimated vaccine effectiveness was 91.8% (95% CI, 77.7−97.0%). In 3 studies evaluating vaccine effectiveness among HCWs with 2 doses,^19,22,30^ the pooled DOR was 0.035 (95% CI, 0.013−0.100) and the estimated vaccine effectiveness was 96.5% (95% CI, 90.0−98.7%). In 6 studies evaluating vaccine effectiveness among HCWs who had received only 1 dose of vaccine,^19,22–25,30^ the pooled DOR was 0.213 (95% CI, 0.040−1.138) and the estimated vaccine effectiveness was 78.7% (95% CI, −13.8% to 96.0%). Stratifying the analysis for only asymptomatic COVID-19, 4 studies evaluated vaccine effectiveness among HCWs who had received 2 doses of vaccine.^19,21,22,30^ The pooled DOR for this group of studies was 0.089 (95% CI, 0.029−0.274) and the estimated vaccine effectiveness was 85.3% (95% CI, 47.7%−95.9%). In 3 studies evaluating vaccine effectiveness among HCWs who had received only 1 dose of vaccine,^19,22,30^ the pooled DOR for this group of studies was 0.364 (95% CI, 0.104−1.276) and the estimated vaccine effectiveness was 63.3% (95% CI, −27.6% to 89.6%).

The results of both meta-analyses were homogeneous for symptomatic COVID-19 (all studies evaluating vaccinated HCWs: heterogeneity *P* = .86, I^2^ = 0%; 2 doses: heterogeneity *P* = 0.70, I^2^ = 0%; 1 dose: heterogeneity *P* = 0.43, I^2^ = 0%). The results were homogeneous for symptomatic and asymptomatic COVID-19 (all studies evaluating vaccinated HCWs: heterogeneity *P* = .78, I^2^ = 0%; 2 doses: heterogeneity *P* = .49, I^2^ = 0%; 1 dose: heterogeneity *P* = .56, I^2^ = 0%). The results were also homogenous for only asymptomatic COVID-19 in 2 doses and 1 dose: heterogeneity *P* = .25, I^2^ = 27.0%; heterogeneity *P* = .43, I^2^ = 0%, respectively. The reasons for not including the other 3 COVID-19 vaccine HCW studies in the meta-analysis are summarized in Supplementary Appendix 2 .

## Discussion

Based on studies evaluating short-term vaccine effectiveness between December 2020 to April 2021, this systematic literature review and meta-analysis showed that COVID-19 vaccines (primarily the mRNA COVID-19 vaccines) decrease symptomatic COVID-19 with a vaccine effectiveness of 92.8%. This number was comparable to vaccine effectiveness among the general population reported in the randomized trials^1,7^ and in a noncontrolled setting.^5^ COVID-19 vaccines were also effective in reducing asymptomatic COVID-19.

Multiple vaccines are being distributed worldwide under emergency use authorizations, and additional vaccine candidates are already in phase 3 studies assessing efficacy.^34^ In our systematic literature review, we were only able to analyze the vaccine effectiveness for the mRNA COVID-19 vaccines (Pfizer/BioNTech and Moderna). These were the first COVID-19 vaccines authorized by the FDA,^35,36^ and HCWs were considered the priority group to receive them.^37^ The short duration of the studies, from 0.5 to 3 months, included in our systematic literature review among HCWs is justified particularly to understand the short-term vaccine effectiveness in the context of a global pandemic with a novel pathogen (Table 1).^34^ This factor also explains the wide confidence intervals (and the negative lower bound) around the vaccine effectiveness of single-dose Pfizer/BioNTech mRNA in our meta-analysis (Table 2).

**Table 2.** Subset Analyses Evaluating the COVID-19 Vaccine Effectiveness among Healthcare Workers (13 studies)^a^

Subset	Studies Included, No.	HCWs, No.	Pooled DOR (95% CI)	I^2^ Test for Heterogeneity, %	Vaccine Effectiveness, % (95% CI)^b^
All studies evaluating vaccinated HCWs (any status)^c^ and symptomatic COVID-19	13	173,742	0.072 (0.028–0.184)	0	92.8% (81.6–97.2)
Studies evaluating 2 doses among HCWs and symptomatic COVID-19	7	97,129	0.065 (0.024–0.175)	0	93.5% (82.5–97.6)
Studies evaluating one dose among HCWs and symptomatic COVID-19	6	103,932	0.179 (0.059–0.539)	0	82.1% (46.1–94.1)
All studies evaluating vaccinated HCWs (any status) and symptomatic and asymptomatic COVID-19	10	158,285	0.082 (0.030–0.223)	0	91.8% (77.7–97.0)
Studies evaluating 2 doses among HCWs and symptomatic and asymptomatic COVID-19	3	84,285	0.035 (0.013–0.100)	0	96.5% (90.0–98.7)
Studies evaluating 1 dose among HCWs and symptomatic and asymptomatic COVID-19	6	137877	0.213 (0.040–1.138)	0	78.7% (−13.8 to 96.0)
Studies evaluating 2 doses among HCWs and asymptomatic COVID-19	4	84,401	0.147 (0.041–0.523)	27	85.3% (47.7–95.9)
Studies evaluating 1 dose among HCWs and asymptomatic COVID-19	3	84,285	0.364 (0.104–1.276)	0	63.6% (−27.6 to 89.6)

Note. DOR, diagnostic odds ratio; HCW, healthcare worker; CI, confidence interval.

a Reasons for not including the other 3 COVID-19 vaccine HCW studies in the meta-analysis: Amit 2021^18^ reported the number of exposure days; Jones 2021^26^ reported the number of positive tests; and Thompson 2021^32^ reported the number of person days. Other reasons for not including studies in the stratified analysis: Bianchi 2021^20^ did not report the total number of HCWs that received the first dose; Hall 2021^25^ reported the number of person days for HCWs that received the second dose; Tang 2021^31^ did not report the total number of HCWs who received the first and the second dose.

b Vaccine effectiveness was estimated as 100% × (1 − DOR).

c Vaccinated HCWs considering any vaccination status (1 dose or 2 doses). If the study reported 2 doses, we have considered the second dose; if the study reported only 1 dose, we have considered the first dose with a longer time (eg, 0–14 days; 14–21; and ≥21 days, the last 1 was selected for the analysis).

Stratified analyses with 4 studies investigating vaccine effectiveness against asymptomatic COVID-19 also revealed high vaccine effectiveness among HCWs with 1 dose and 2 doses: 63.6% and 85.3%, respectively.^19,21,22,30^ Given that most SARS-CoV-2 is transmitted by asymptomatic individuals or prior to symptom onset in symptomatic individuals, COVID-19 vaccines might have a bigger role in preventing SARS-CoV-2 transmission than is recognized currently with reported symptomatic cases.^37,38^ Symptomatic COVID-19 is well recognized, and individuals with COVID-19 symptoms are more likely to isolate themselves, which further reduces the proportion of transmission from symptomatic individuals. The knowledge that COVID-19 vaccines are effective even in asymptomatic people could contribute to substantially reducing the transmission of SARS-CoV-2 and controlling the COVID-19 pandemic.^38,39^

Only 1 study reported an R.1 lineage variant.^21^ This study was conducted in a nursing facility after a vaccination program and showed that vaccinated HCWs were 87% less likely to have symptomatic COVID-19 than those who were unvaccinated. Also, 2 studies performed genomic surveillance detecting the B.1.1.7 variant or α variant.^26,29^ The other studies did not include genomic surveillance. Hall et al^25^ reported that the HCW cohort was vaccinated when the dominant variant in circulation was B1.1.7 and showed effectiveness against this variant. Our systematic review included studies prior to the widespread circulation of the delta variant, which has contributed to most recent breakthrough infections among HCWs.^40,41^ More studies are needed regarding the SARS-CoV-2 variants of concerns (VOC) that have multiple spike protein mutations and that appear to be more infectious or cause more disease than other circulating SARS-CoV-2 variants.^42^ Some deletions in the spike protein mutations can alter the shape of the spike and may help it evade some antibodies.^43^ No COVID-19 vaccine is 100% effective against SARS-CoV-2 infection, which is consistent with COVID-19 breakthrough infections reported among HCWs after COVID-19 vaccination.^44,45^

Our study had several limitations. We only included observational studies for the meta-analysis, which are subject to multiple biases^46^; however, this is the most common study design in the infection prevention literature.^46^ We could not investigate vaccine effectiveness of other COVID-19 vaccines due to lack of published studies. We estimated the vaccine effectiveness based on only short-term study durations, and longer-term observational studies are needed to assess sustained immune response and vaccine effectiveness. Due to the uncertainty related to the number of days required to develop immunity postvaccination, each study adopted a different definition of a fully vaccinated or partially vaccinated person. The CDC defines people fully vaccinated as being ≥14 days after the second dose in a 2-dose series (Pfizer/BioNTech or Moderna) or ≥14 days after a single dose vaccine (Johnson & Johnson/Janssen).^47^ Currently, no postvaccination time limit on fully vaccinated status has been established. In addition, the CDC defines unvaccinated people as individuals of all ages, including children who have not completed a vaccination series or received a single-dose vaccine.^47^ No consensus had been reached regarding fully vaccinated versus partially vaccinated in the included studies, and the studies used different criteria (eg, fully vaccinated for ≥7–14 days after the second dose, partially vaccinated for ≥14 days after the first dose, or just reporting the first dose available).^18–33^ None of the included studies reported information about possible adverse events after vaccine administration. For that reason, we were not able to report any evidence of severe complications and we were unable to assess whether vaccinated HCWs sought further COVID-19 testing. We could not perform further analyses stratified by immunocompromised status due to the limited studies available. We did not investigate the association between vaccine effectiveness and personal protective equipment, although vaccine effectiveness might have been affected by the PPE recommended at each institution. Because our study focused on only the short-term vaccine effectiveness among HCWs, we did not evaluate the need for the third dose. Lastly, each study used a different approach to reporting the incidence of COVID-19 (eg, incidence rate per person days and per exposure days). Therefore, we performed our meta-analysis and stratified analyses using a bivariate approach to preserve the 2-dimensional nature of the original data from the selected studies.^19–25,27–30,33^

In conclusion, the COVID-19 mRNA vaccines can significantly prevent symptomatic and asymptomatic COVID-19 among HCWs. The COVID-19 vaccines are also effective among HCWs, even after 1 dose. These data are very important for countries struggling to offer COVID-19 vaccines for HCWs because of limited resources. To better understand vaccine effectiveness against the new SARS-CoV-2 variants, more observational studies are needed to evaluate (1) other types of COVID-19 vaccine (eg, viral vector or inactivated virus) effectiveness, (2) the impact of personal protective equipment among HCWs on vaccine effectiveness, (3) COVID-19 breakthrough after vaccination, and (4) genomic surveillance.

## Supplementary material

For supplementary material accompanying this paper visit https://doi.org/10.1017/ash.2021.195. click here to view supplementary material

## References

[1] Polack FP , Thomas SJ , Kitchin N , et al. Safety and efficacy of the BNT162b2 mRNA COVID-19 vaccine. N Engl J Med 2020;383:2603–2615. 33301246 10.1056/NEJMoa2034577 PMC7745181

[2] Hodgson SH , Mansatta K , Mallett G , Harris V , Emary KRW , Pollard AJ. What defines an efficacious COVID-19 vaccine? A review of the challenges assessing the clinical efficacy of vaccines against SARS-CoV-2. Lancet Infect Dis 2021;21(2):e26–e35. 33125914 10.1016/S1473-3099(20)30773-8 PMC7837315

[3] Mutambudzi M , Niedwiedz C , Macdonald EB , et al. Occupation and risk of severe COVID-19: prospective cohort study of 120 075 UK Biobank participants. *Occup Environ Med* 2020. doi: 10.1136/oemed-2020-106731. 10.1136/oemed-2020-106731 PMC7611715 33298533

[4] Nguyen LH , Drew DA , Graham MS , et al. Risk of COVID-19 among frontline healthcare workers and the general community: a prospective cohort study. Lancet Public Health 2020;5(9):e475–e483. 32745512 10.1016/S2468-2667(20)30164-X PMC7491202

[5] Dagan N , Barda N , Kepten E , et al. BNT162b2 mRNA COVID-19 vaccine in a nationwide mass vaccination setting. N Engl J Med 2021;384:1412–1423. 33626250 10.1056/NEJMoa2101765 PMC7944975

[6] Tenforde MW , Olson SM , Self WH , et al. Effectiveness of Pfizer-BioNTech and Moderna vaccines against COVID-19 among hospitalized adults aged ≥65 years—United States, January–March 2021. Morb Mortal Wkly Rep 2021;70:674–679. 10.15585/mmwr.mm7018e1 PMC9368749 33956782

[7] Baden LR , El Sahly HM , Essink B , et al. Efficacy and safety of the mRNA-1273 SARS-CoV-2 vaccine. N Engl J Med 2021;384:403–416. 33378609 10.1056/NEJMoa2035389 PMC7787219

[8] Benenson S , Oster Y , Cohen MJ , Nir-Paz R. BNT162b2 mRNA COVID-19 vaccine effectiveness among healthcare workers. N Engl J Med 2021;384:1775–1777. 33755373 10.1056/NEJMc2101951 PMC8008751

[9] Abu Jabal K , Ben-Amram H , Beiruti K , et al. Impact of age, ethnicity, sex, and prior infection status on immunogenicity following a single dose of the BNT162b2 mRNA COVID-19 vaccine: real-world evidence from healthcare workers, Israel, December 2020 to January 2021. Euro Surveill 2021;26:2100096. 10.2807/1560-7917.ES.2021.26.6.2100096 PMC7879501 33573712

[10] Moher D , Liberati A , Tetzlaff J , Altman DG , The PRISMA Group. Preferred reporting items for systematic reviews and meta-analyses: the PRISMA statement. PloS Med 2009;6:e1000097. 19621072 10.1371/journal.pmed.1000097 PMC2707599

[11] Stroup DF , Berlin JA , Morton SC , et al. Meta-analysis of observational studies in epidemiology: a proposal for reporting. Meta-analysis of Observational Studies in Epidemiology (MOOSE) group. JAMA 2000;283:2008–2012. 10789670 10.1001/jama.283.15.2008

[12] US Department of Health and Human Services, Center for Biologics Evaluation and Research. Development and licensure of vaccines to prevent COVID-19: guidance for industry. US Food and Drug Administration website. www.fda.gov/media/139638/download. Accessed June 6, 2021.

[13] Downs SH , Black N. The feasibility of creating a checklist for the assessment of the methodological quality both of randomized and nonrandomised studies of health care interventions. J Epidemiol Commun Health 1998;52:377–384. 10.1136/jech.52.6.377 PMC1756728 9764259

[14] Alderson PGS , Higgins JPT , editors. Assessment of study quality. Cochrane Reviewer’s Handbook 4.2.3. Chichester, UK: John Wiley & Sons; 2004.

[15] Doebler P. Meta-analysis of diagnostic accuracy with mada. R package version 0.5.8. https://cran.r-project.org/web/packages/mada/vignettes/mada.pdf. Published 2017. Accessed June 4, 2021.

[16] Reitsma JB , Glas AS , Rutjes AW , Scholten RJ , Bossuyt PM , Zwinderman AH. Bivariate analysis of sensitivity and specificity produces informative summary measures in diagnostic reviews. J Clin Epidemiol 2005;58:982–990. 16168343 10.1016/j.jclinepi.2005.02.022

[17] Goto M , Ohl ME , Schweizer ML , Perencevich EN. Accuracy of administrative code data for the surveillance of healthcare-associated infections: a systematic review and meta-analysis. Clin Infect Dis 2014;58:688–696. 24218103 10.1093/cid/cit737

[18] Amit S , Regev-Yochay G , Afek A , Kreiss Y , Leshem E. Early rate reductions of SARS-CoV-2 infection and COVID-19 in BNT162b2 vaccine recipients. Lancet 2021;397:875–877. 10.1016/S0140-6736(21)00448-7 PMC7906709 33610193

[19] Angel Y , Spitzer A , Henig O , et al. Association between vaccination with BNT162b2 and incidence of symptomatic and asymptomatic SARS-CoV-2 infections among healthcare workers. *JAMA* 2021. doi: 10.1001/jama.2021.7152. 10.1001/jama.2021.7152 PMC8220476 33956048

[20] Bianchi FP , Germinario CA , Migliore G , et al. BNT162b2 mRNA COVID-19 vaccine effectiveness in the prevention of SARS-CoV-2 infection: a preliminary report. *J Infect Dis* 2021. doi: 10.1093/infdis/jiab262. 10.1093/infdis/jiab262 PMC8194590 34007998

[21] Cavanaugh AM , Fortier S , Lewis P , et al. COVID-19 outbreak associated with a SARS-CoV-2 R.1 lineage variant in a skilled nursing facility after vaccination program—Kentucky, March 2021. Morb Mortal Wkly Rep 2021;70:639–643. 10.15585/mmwr.mm7017e2 PMC8084128 33914720

[22] Fabiani M , Ramigni M , Gobbetto V , Mateo-Urdiales A , Pezzotti P , Piovesan C. Effectiveness of the Comirnaty (BNT162b2, BioNTech/Pfizer) vaccine in preventing SARS-CoV-2 infection among healthcare workers, Treviso province, Veneto region, Italy, 27 December 2020 to 24 March 2021. *Euro Surveill* 2021. doi: 10.2807/1560-7917.ES.2021.26.17.2100420. 10.2807/1560-7917.ES.2021.26.17.2100420 PMC8086247 33928898

[23] Garvey MI , Wilkinson MAC , Holden E , et al. Early observations on the impact of a healthcare worker COVID-19 vaccination programme at a major UK tertiary centre. *J Infect* 2021. doi: 10.1016/j.jinf.2021.04.027. 10.1016/j.jinf.2021.04.027 PMC8081749 33933530

[24] Gras-Valentí P , Chico-Sánchez P , Algado-Sellés N , et al. Efectividad de la primera dosis de vacuna BNT162b2 para prevenir la COVID-19 en personal sanitario [Effectiveness of the first dose of BNT162b2 vaccine to preventing COVID-19 in healthcare personnel]. Rev Esp Salud Publica 2021;95:e202104070. 33913444

[25] Hall VJ , Foulkes S , Saei A , et al. COVID-19 vaccine coverage in healthcare workers in England and effectiveness of BNT162b2 mRNA vaccine against infection (SIREN): a prospective, multicentre, cohort study. Lancet 2021;397:1725–1735. 33901423 10.1016/S0140-6736(21)00790-X PMC8064668

[26] Jones NK , Rivett L , Seaman S , et al. Single-dose BNT162b2 vaccine protects against asymptomatic SARS-CoV-2 infection. Elife 2021;10:e68808. 33830018 10.7554/eLife.68808 PMC8064747

[27] Pilishvili T , Fleming-Dutra KE , Farrar JL , et al. Interim estimates of vaccine effectiveness of Pfizer-BioNTech and Moderna COVID-19 vaccines among healthcare personnel—33 US sites, January–March 2021. Morb Mortal Wkly Rep 2021;70:753–758. 10.15585/mmwr.mm7020e2 PMC8136422 34014909

[28] Pryor R , Cooper K , Britton A , et al. Riding the third wave: how an academic medical center reduced COVID-19 infection in healthcare workers. *N Engl J Med* website. https://catalyst.nejm.org/doi/pdf/10.1056/CAT.21.0060. Accessed May 31, 2021.

[29] Sansone E , Tiraboschi M , Sala E , et al. Effectiveness of BNT162b2 vaccine against the B.1.1.7 variant of SARS-CoV-2 among healthcare workers in Brescia, Italy. *J Infect* 2021. doi: 10.1016/j.jinf.2021.04.038. 10.1016/j.jinf.2021.04.038 PMC8102078 33965428

[30] Swift MD , Breeher LE , Tande AJ , et al. Effectiveness of mRNA COVID-19 vaccines against SARS-CoV-2 infection in a cohort of healthcare personnel. *Clin Infect Dis* 2021. doi: 10.1093/cid/ciab361. 10.1093/cid/ciab361 PMC8135611 33900384

[31] Tang L , Hijano DR , Gaur AH , et al. Asymptomatic and symptomatic SARS-CoV-2 infections after BNT162b2 vaccination in a routinely screened workforce. JAMA 2021;325:2500–2502. 33956050 10.1001/jama.2021.6564 PMC8220512

[32] Thompson MG , Burgess JL , Naleway AL , et al. Interim estimates of vaccine effectiveness of BNT162b2 and mRNA-1273 COVID-19 vaccines in preventing SARS-CoV-2 infection among healthcare personnel, first responders, and other essential and frontline workers—eight US locations, December 2020–March 2021. Morb Mortal Wkly Rep 2021;70:495–500. 10.15585/mmwr.mm7013e3 PMC8022879 33793460

[33] Walsh J , Skally M , Traynor L , et al. Impact of first dose of BNT162b2 vaccine on COVID-19 infection among healthcare workers in an Irish hospital. *Ir J Med Sci* 2021. doi: 10.1007/s11845-021-02658-4. 10.1007/s11845-021-02658-4 PMC8154332 34041693

[34] Hodgson SH , Mansatta K , Mallett G , Harris V , Emary KRW , Pollard AJ. What defines an efficacious COVID-19 vaccine? A review of the challenges assessing the clinical efficacy of vaccines against SARS-CoV-2. Lancet Infect Dis 2021;21(2):e26–e35. 33125914 10.1016/S1473-3099(20)30773-8 PMC7837315

[35] Pfizer/BioNTech COVID-19 vaccine. US Food and Drug Administration website. https://www.fda.gov/emergency-preparedness-and-response/coronavirus-disease-2019-covid-19/pfizer-biontech-covid-19-vaccine. Accessed June 6, 2021.

[36] Moderna COVID-19 vaccine. US Food and Drug Administration website. https://www.fda.gov/emergency-preparedness-and-response/coronavirus-disease-2019-covid-19/moderna-covid-19-vaccine. Accessed June 6, 2021.

[37] Mehrotra DV , Janes HE , Fleming TR , et al. Clinical end points for evaluating efficacy in COVID-19 vaccine trials. Ann Intern Med 2021;174:221–228. 33090877 10.7326/M20-6169 PMC7596738

[38] He X , Lau EHY , Wu P , et al. Temporal dynamics in viral shedding and transmissibility of COVID-19. Nat Med 2020;26:672–675. 32296168 10.1038/s41591-020-0869-5

[39] Johansson MA , Quandelacy TM , Kada S , et al. SARS-CoV-2 transmission from people without COVID-19 symptoms. JAMA Netw Open 2021;4(1):e2035057. 33410879 10.1001/jamanetworkopen.2020.35057 PMC7791354

[40] del Rio C , Malani PN , Omer SB. Confronting the delta variant of SARS-CoV-2, summer 2021. *JAMA* 2021. doi:10.1001/jama.2021.14811. 10.1001/jama.2021.14811 34406361

[41] Lustig Y , Zuckerman N , Nemet I , et al. Neutralising capacity against delta (B.1.617.2) and other variants of concern following Comirnaty (BNT162b2, BioNTech/Pfizer) vaccination in health care workers, Israel. *Euro Surveill* 2021. doi: 10.2807/1560-7917.ES.2021.26.26.2100557. 10.2807/1560-7917.ES.2021.26.26.2100557 PMC8326656 34212838

[42] Challen R , Brooks-Pollock E , Read JM , Dyson L , Tsaneva-Atanasova K , Danon L. Risk of mortality in patients infected with SARS-CoV-2 variant of concern 202012/1: matched cohort study. BMJ 2021;372:n579. 33687922 10.1136/bmj.n579 PMC7941603

[43] Wang P , Nair MS , Liu L , et al. Antibody resistance of SARS-CoV-2 variants B.1.351 and B.1.1.7. Nature 2021;593:130–135. 33684923 10.1038/s41586-021-03398-2

[44] Hacisuleyman E , Hale C , Saito Y , et al. Vaccine breakthrough infections with SARS-CoV-2 Variants. *N Engl J Med* 2021. doi: 10.1056/NEJMoa2105000. 10.1056/NEJMoa2105000 PMC8117968 33882219

[45] CDC COVID-19 Vaccine Breakthrough Case Investigations Team. COVID-19 vaccine breakthrough infections reported to CDC—United States, January 1–April 30, 2021. Morb Mortal Wkly Rep 2021;70:792–793. 10.15585/mmwr.mm7021e3 PMC8158893 34043615

[46] Harris AD , Lautenbach E , Perencevich E. A systematic review of quasi-experimental study designs in the fields of infection control and antibiotic resistance. Clin Infect Dis 2005;41:77–82. 15937766 10.1086/430713

[47] Interim Public Health recommendations for fully vaccinated people. COVID-19 Centers for Disease Control and Prevention website. https://www.cdc.gov/coronavirus/2019-ncov/vaccines/fully-vaccinated-guidance.html. Accessed June 18, 2021.

