# Supplementary material for: The short-term effectiveness of coronavirus disease 2019 (COVID-19) vaccines among healthcare workers: a systematic literature review and meta-analysis
Source: Antimicrob Steward Healthc Epidemiol. 2021 Oct 21;1(1):e33. doi: 10.1017/ash.2021.195 (PMC9495770; doi:10.1017/ash.2021.195)

| **Supplementary Appendix 1.** Search terms and strategies | | |
| --- | --- | --- |
| **Vaccines** | **COVID 19** | **Health care personnel** |
| Medical subject headings (PubMed)  Covid-19 Vaccines  RNA, Messenger  Vaccines  Vaccination  Immunization | Medical subject headings (PubMed)  COVID-19  SARS-CoV-2  Coronavirus Infections (no explode) | Medical subject headings (PubMed)  Health personnel (exploded) |
| Emtree (Embase)  SARS CoV-2 Vaccine  Messenger RNA  SARS-CoV-2 antibody  Vaccine  Vaccination  Immunization | Emtree (Embase)  Coronavirus disease 2019  Coronavirus infection (no explode) | Emtree (Embase)  Health care personnel (exploded) |
| CINAHL headings  COVID 19 vaccines  RNA, Messenger  Vaccines  Immunization (no explode, includes anti vacc movement and vacc rates) | CINAHL headings  COVID-19  SARS-CoV-2  Coronavirus infections (no explode) | CINAHL headings  Health personnel (exploded) |
| Free text words/phrases  Vaccine(s)  Vaccination(s)  Pfizer  Bnt162b2  Moderna  immunization(s)  variolation(s)  Immunologic stimulation  Immunostimulation  mRNA-1273  ChAdOx1-S  AZD1222  AstraZeneca/Oxford  Janssen  Johnson&Johnson  Ad26COVS1  JNJ-78436735  Gamaleya  Sputnik V  Sinovac  Corona Vac | Free text words/phrases  Covid  Covid 19  Corona virus  Coronavirus  2019-nCoV  SARS-CoV-2  2019-nCoV | Free text words/phrases  health care personnel  Healthcare personnel  Health care employee(s)  Healthcare employee(s)  Health care worker(s)  Healthcare worker(s)  Hospital staff  Doctor(s)  Physician(s)  Nurs (ing, e, es)  Therapist(s)  Psychologist(s)  Dentist(s)  Pharmacist(s)  Optometrist(s)  Nutritionist(s)  Case manager(s)  Anesthesiologist(s)  Anesthetist(s)  Audiologist(s)  Coroner(s)  Home health  Physician assistant(s)  Radiologist(s)  Oncologist(s)  Ophthalmologist(s)  Pediatrician(s)  Pulmonologist(s)  Rheumatologist (s)  surgeon(s)  Urologist(s)  Community health worker (s)  Operating room Technician(s)  Laboratory personnel  Medical technologist(s)  Laboratory technician(s)  Allied health personnel  X ray technician(s)  Emergency medical  Paramedic(s)  clinician(s)  practitioner(s)  Health care professional(s)  Healthcare professional(s) |

**PubMed search strategy, 6/11/21 (NLM interface)**

#1

"COVID-19 Vaccines"[Mesh] AND "Health Personnel"[Mesh]

#2

"RNA, Messenger"[Mesh] OR "Vaccines"[Mesh] OR "Vaccination"[Mesh] OR "Immunization"[Mesh] OR vaccine[Text Word] OR vaccines[Text Word] OR vaccination[Text Word] OR vaccinations[Text Word] OR immunization[Text Word] OR immunizations[Text Word] OR variolation[Text Word] OR variolations[Text Word] OR immunologic stimulation[Text Word] OR immunostimulation[Text Word] OR pfizer[Text Word] OR bnt162b2 [Text Word] OR moderna[Text Word] OR ChAdOx1-S [Text Word] OR AZD1222 [Text Word] OR AstraZeneca [Text Word] OR Oxford [Text Word] OR Janssen [Text Word] OR Johnson [Title/Abstract] OR Ad26COVS1 [Text Word] OR JNJ-78436735 [Text Word] OR Gamaleya [Text Word] OR  Sputnik V [Text Word] OR Sinovac [Text Word] OR Corona Vac [Text Word]

#3

"COVID-19"[Mesh] OR "SARS-CoV-2"[Mesh] OR "Coronavirus Infections"[Mesh:NoExp] OR Covid[Text Word] OR Covid 19[Text Word] OR coronavirus[Text Word] OR corona virus[Text Word] OR 2019 ncov[Text Word] OR sars cov 2[Text Word] OR 2019 nCov[Text Word]

#4

"Health Personnel"[Mesh] OR health care personnel[Text Word] OR Healthcare personnel [Text Word] OR Health care employee[Text Word] OR health care employees[Text Word] OR healthcare employees[Text Word] OR Healthcare employee[Text Word] OR Health care worker[Text Word] OR Health care workers[Text Word] OR Healthcare worker[Text Word] OR Healthcare workers[Text Word] OR health care professionals [Text Word] OR health care professional [Text Word] OR clinician [Text Word] OR clinicians [Text Word] OR hospital staff[Text Word] OR doctor[Text Word] OR doctors[Text Word] OR physicians[Text Word] OR physician[Text Word] OR nursing[Text Word] OR nurses[Text Word] OR nurse[Text Word] OR therapist[Text Word] OR therapists[Text Word] OR psychologist[Text Word] OR psychologists[Text Word] OR dentist[Text Word] OR dentists[Text Word] OR pharmacist[Text Word] OR pharmacists[Text Word] OR optometrist[Text Word] OR optometrists[Text Word] OR nutritionist[Text Word] OR nutritionists[Text Word] OR case manager[Text Word] OR case managers[Text Word] OR anesthesiologist[Text Word] OR anesthesiologists[Text Word] OR anesthetist[Text Word] OR anesthetists[Text Word] OR audiologists[Text Word] OR audiologist[Text Word] OR coroner[Text Word] OR coroners[Text Word] OR home health[Text Word] OR physician assistant[Text Word] OR physician assistants[Text Word] OR radiologist[Text Word] OR radiologists[Text Word] OR oncologist[Text Word] OR oncologists[Text Word] OR ophthalmologist[Text Word] OR opthalmologists[Text Word] OR pediatrician[Text Word] OR pediatricians[Text Word] OR pulmonologist[Text Word] OR pulmonologists[Text Word] OR rheumatologist[Text Word] OR rheumatologists[Text Word] OR surgeon[Text Word] OR surgeons[Text Word] OR urologist[Text Word] OR urologists[Text Word] OR community health worker[Text Word] OR community health workers[Text Word] OR operating room technician[Text Word] OR operating room technicians[Text Word] OR laboratory personnel[Text Word] OR medical technologists[Text Word] OR medical technologist[Text Word] OR laboratory technician[Text Word] OR laboratory technicians[Text Word] OR allied health personnel[Text Word] OR x ray technician[Text Word] OR x ray technicians[Text Word] OR emergency medical[Text Word] OR paramedic[Text Word] OR paramedics[Text Word] OR hospitalist[Text Word] OR hospitalists[Text Word] OR practitioner [Text Word] OR practitioners [Text Word]

#4

 "Animals"[MeSH Terms] NOT ("Animals"[MeSH Terms] AND "Humans"[MeSH Terms])

OR

(hesitance[Title] OR hesitancy[Title] OR hesitation [Title] OR attitude[Title] OR attitudes[Title] OR acceptance[Title] OR compliance[Title] OR accept [Title] OR acceptability [TItle] OR perception [TItle] OR perceptions [Title] OR influence [TItle] OR influencing [TItle] OR intent [Title] OR intention [Title] OR intentions [Title] OR perspective [Title] OR perspectives [TItle] OR opinion [Title] OR opinions [Title] OR willingness [Title] OR receptivity [TItle] OR barriers [Title] OR barrier [TItle] OR motivation [TItle] OR behavior [TItle] OR belief [Title] OR beliefs [Title])

#1 OR (#2 AND #3 AND #4) NOT #4= 1149

**CINAHL search strategy, 6/11/21 (EBSCO interface)**

#1

MH "COVID-19 Vaccines" AND MH "Health Personnel+"

#2

(MH "RNA, Messenger" OR MH "Vaccines+" OR MH "Immunization" OR Vaccine* OR Vaccination* OR  Pfizer OR  Moderna OR immunization* OR variolation* OR “immunologic stimulation" OR  Immunostimulation OR Gamaleya OR "Sputnik V" OR  Sinovac OR "Corona Vac" OR AstraZeneca OR Janssen OR "AZD1222" OR "mRNA-1273" OR Janssen OR "Johnson & Johnson" OR "JNJ-78436735")

AND

(MH "Coronavirus Infections" OR MH "COVID-19" OR MH "SARS-CoV-2" OR Covid OR "Covid 19" OR "Corona virus" OR Coronavirus OR "2019-nCoV" OR "SARS-CoV-2" OR   "2019-nCoV")

AND

(MH "Health Personnel+" OR “health care personnel*”  OR “Health care employee*” OR “Healthcare employee” OR “Health care worker*” OR “Healthcare worker*” OR  “health care professional*” OR healthcare professional*” OR “health professional*” OR clinician* OR “hospital staff” OR doctor* OR physician* OR nurs* OR therapist* OR psychologist* OR dentist* OR pharmacist*  OR optometrist* OR nutritionist* OR “case manager*” OR anesthesiologist* OR anesthetist* OR audiologist* OR pathologist* OR  coroner* OR home health OR physician assistant* OR radiologist* OR oncologist* OR neurologist* OR endocrinologist* OR podiatrist*  OR dermatologist* OR ophthalmologist* OR pediatrician* OR pulmonologist* OR rheumatologist* OR surgeon* OR urologist* OR “community health worker*” OR “operating room technician*” OR “laboratory personnel” OR “medical technologists” OR “medical technologist*” OR “laboratory technician*” OR “allied health personnel*” OR “x ray technician*” OR “emergency medical” OR paramedic* OR hospitalist* OR practitioner*)

#3

TI (hesitance OR hesitancy OR hesitation  OR attitude OR attitudes OR acceptance OR compliance OR accept  OR acceptability  OR perception  OR perceptions  OR influence  OR influencing  OR intent  OR intention  OR intentions  OR perspective  OR perspectives  OR opinion  OR opinions  OR willingness  OR receptivity  OR barriers  OR barrier  OR motivation  OR behavior* OR belief*) OR PT (commentary OR editorial OR pictorial OR brief OR letter)

(#1 OR #2) NOT #3=133

**Scopus search strategy, 6/10/21 (Elsevier interface)**

((TITLE-ABS-KEY(Vaccine* OR Vaccination* OR Pfizer OR Moderna OR immunization* OR variolation* OR "immunologic stimulation") OR TITLE-ABS-KEY(Immunostimulation OR Gamaleya OR "Sputnik V" OR Sinovac OR "Corona Vac" OR AstraZeneca OR Janssen OR "AZD1222" OR "mRNA-1273" OR Janssen OR "Johnson & Johnson" OR "JNJ-78436735")) AND ((TITLE-ABS-KEY(covid OR "Covid 19" OR "Corona virus" OR Coronavirus OR 2019nCoV OR SARSCoV2 OR 2019-nCoV)) AND ((TITLE-ABS-KEY(“health care personnel” OR “Health care employee” OR “Health care employees” OR “Healthcare employee” OR “Healthcare employees” OR “Health care worker” OR “Health care workers” OR “Healthcare worker” OR "healthcare workers") OR TITLE-ABS-KEY(“health care professional” OR "health care professionals" OR healthcare professional” OR "healthcare professionals" OR clinician* OR "health professional" OR "health professionals") OR TITLE-ABS-KEY("hospital staff" OR doctor* OR physician* OR nurs* OR therapist* OR psychologist* OR dentist* OR pharmacist* OR optometrist* OR nutritionist*) OR TITLE-ABS-KEY("case manager" OR "case managers" OR anesthesiologist* OR anesthetist* OR audiologist* OR pathologist* OR coroner* OR "home health" OR "physician assistant" OR "physician assistants") OR TITLE-ABS-KEY(oncologist* OR neurologist* OR endocrinologist* OR podiatrist* OR dermatologist* OR ophthalmologist* OR pediatrician* OR pulmonologist* OR rheumatologist* OR surgeon* OR urologist*) OR TITLE-ABS-KEY(“community health worker” OR "community health workers" OR “operating room technician” OR "operating room technicians" OR “laboratory personnel” OR “medical technologists” OR “medical technologist”) OR TITLE-ABS-KEY(“laboratory technician” OR "laboratory technicians" OR “allied health personnel” OR “x ray technician” OR "x ray technicians" OR “emergency medical” OR paramedic* OR hospitalist* OR practitioner*))))) AND NOT (TITLE(hesitance OR hesitancy OR hesitation OR attitude OR attitudes OR acceptance OR compliance OR accept OR acceptability OR perception OR perceptions OR influence OR influencing OR intent OR intention OR intentions OR perspective OR perspectives OR opinion OR opinions OR willingness OR receptivity OR barriers OR barrier OR motivation OR behavior* OR belief*))

= 1021 after excluding editorials and book chapters (letters, notes, retained)

**Web of Science search strategy, 6/11/21**

**
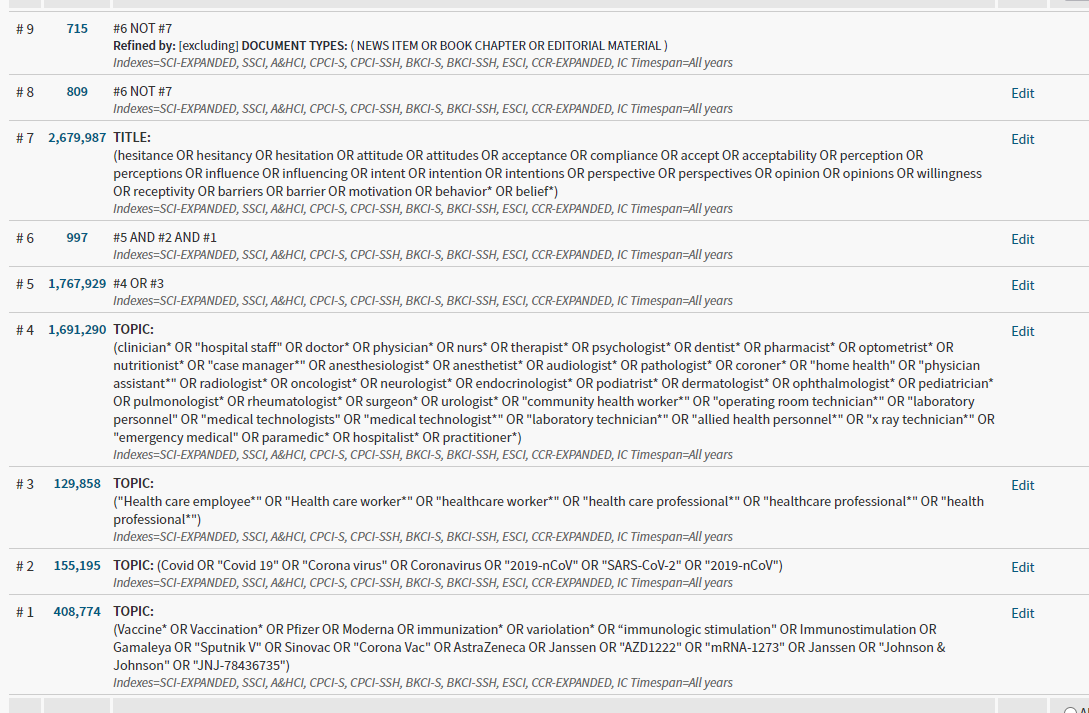
**

**Embase search strategy, 6/11/21 (Elsevier interface)**

#1

'sars-cov-2 vaccine'/exp AND 'health care personnel'/exp

#2

('messenger rna'/exp OR 'sars-cov-2 antibody'/exp OR 'vaccine'/exp OR 'vaccination'/exp OR 'immunization'/exp OR vaccine*:ab,ti OR vaccination*:ab,ti OR pfizer:ab,ti OR moderna:ab,ti OR immunization*:ab,ti OR variolation*:ab,ti OR 'immunologic stimulation':ab,ti OR immunostimulation:ab,ti OR gamaleya:ab,ti OR 'sputnik v':ab,ti OR sinovac:ab,ti OR 'corona vac':ab,ti OR astrazeneca:ab,ti OR 'azd1222':ab,ti OR 'mrna-1273':ab,ti OR janssen:ab,ti OR 'johnson & johnson':ab,ti OR 'jnj-78436735':ab,ti)

AND

('coronavirus disease 2019'/exp OR 'coronavirus infection'/de OR covid:ab,ti OR 'covid 19':ab,ti OR 'corona virus':ab,ti OR coronavirus:ab,ti OR 'sars-cov-2':ab,ti OR '2019-ncov':ab,ti)

AND

('health care personnel'/exp OR 'health care employee*':ab,ti OR 'healthcare employee':ab,ti OR 'health care worker*':ab,ti OR 'healthcare worker*':ab,ti OR 'health care professional*':ab,ti OR 'healthcare professional*':ab,ti OR 'health professional*':ab,ti OR clinician*:ab,ti OR 'hospital staff':ab,ti OR doctor*:ab,ti OR physician*:ab,ti OR nurs*:ab,ti OR therapist*:ab,ti OR psychologist*:ab,ti OR dentist*:ab,ti OR pharmacist*:ab,ti OR optometrist*:ab,ti OR nutritionist*:ab,ti OR 'case manager*':ab,ti OR anesthesiologist*:ab,ti OR anesthetist*:ab,ti OR audiologist*:ab,ti OR pathologist*:ab,ti OR coroner*:ab,ti OR 'home health':ab,ti OR 'physician assistant*':ab,ti OR radiologist*:ab,ti OR oncologist*:ab,ti OR neurologist*:ab,ti OR endocrinologist*:ab,ti OR podiatrist*:ab,ti OR dermatologist*:ab,ti OR ophthalmologist*:ab,ti OR pediatrician*:ab,ti OR pulmonologist*:ab,ti OR rheumatologist*:ab,ti OR surgeon*:ab,ti OR urologist*:ab,ti OR 'community health worker*':ab,ti OR 'operating room technician*':ab,ti OR 'laboratory personnel':ab,ti OR 'medical technologists':ab,ti OR 'medical technologist*':ab,ti OR 'laboratory technician*':ab,ti OR 'allied health personnel*':ab,ti OR 'x ray technician*':ab,ti OR 'emergency medical':ab,ti OR paramedic*:ab,ti OR hospitalist*:ab,ti OR  practitioner':ab,ti)

#3

hesitance:ti OR hesitancy:ti OR hesitation:ti OR attitude:ti OR attitudes:ti OR acceptance:ti OR compliance:ti OR accept:ti OR acceptability:ti OR perception:ti OR perceptions:ti OR influence:ti OR influencing:ti OR intent:ti OR intention:ti OR intentions:ti OR perspective:ti OR perspectives:ti OR opinion:ti OR opinions:ti OR willingness:ti OR receptivity:ti OR barriers:ti OR barrier:ti OR motivation:ti OR behavior*:ti OR belief*:ti

OR

 ('chapter'/it OR 'editorial'/it)

(#1 OR #2) NOT #3= 2120

**Cochrane Trials 6/11/21 (Wiley interface)**


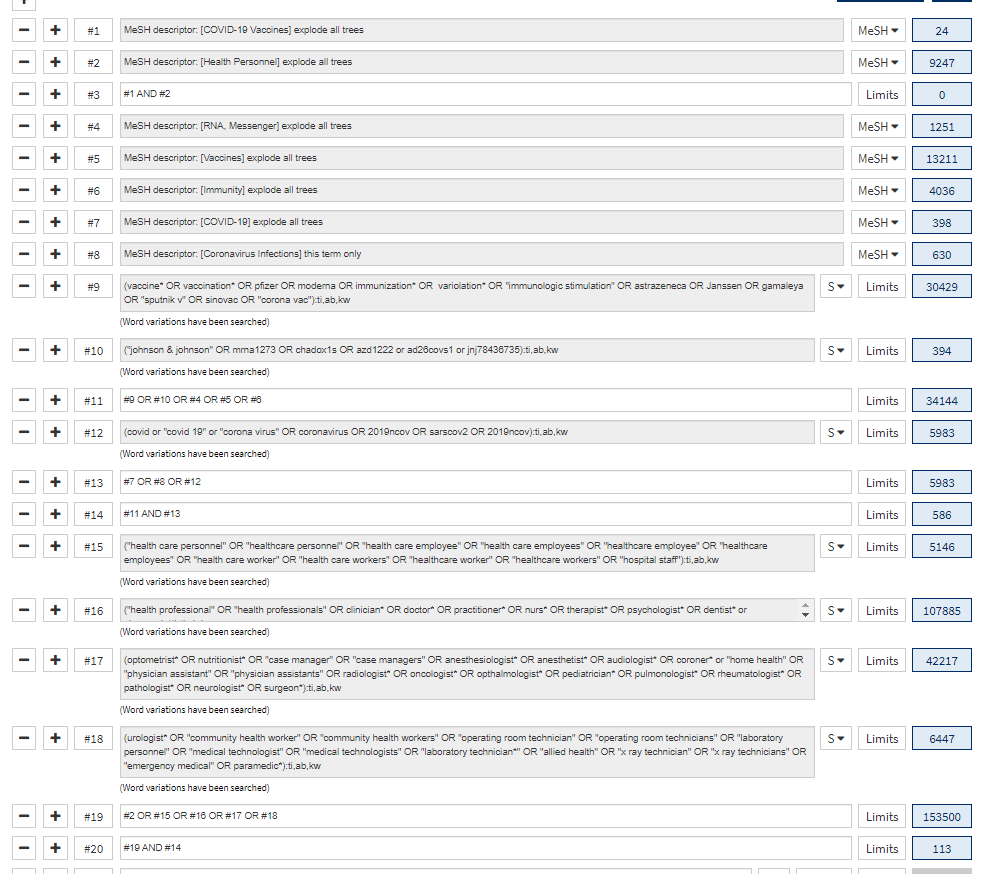

Supplement: Supplementary file 1 [file ashsup.zip › S2732494X21001959sup001.docx]
