# Supplementary material for: The short-term effectiveness of coronavirus disease 2019 (COVID-19) vaccines among healthcare workers: a systematic literature review and meta-analysis
Source: Antimicrob Steward Healthc Epidemiol. 2021 Oct 21;1(1):e33. doi: 10.1017/ash.2021.195 (PMC9495770; doi:10.1017/ash.2021.195)
Supplement: Supplementary file 1 [file ashsup.zip › S2732494X21001959sup002.docx]

**Supplementary Appendix 2**. Reasons for non-inclusion in meta-analysis

The reasons for not including the other 3 COVID-19 vaccine HCWs’ studies in the meta-analysis are: Amit 2021 reported the number of exposure-days [18]; Jones 2021 reported the number of positive tests [26]; Thompson 2021 reported the number of person-days [32]. Also, other reasons for not including studies in the stratified analysis: Bianchi 2021 did not report the total number of HCWs that received the first dose [20]; Hall 2021 reported the number of person-time (days) for HCWs that received the second dose [25]; Tang 2021 did not report the total number of HCWs that received the first and the second dose [31].
